# Association of Dietary Fiber Intake With Myocardial Infarction and Stroke Events in US Adults: A Cross-Sectional Study of NHANES 2011–2018

**DOI:** 10.3389/fnut.2022.936926

**Published:** 2022-06-21

**Authors:** Weiwei Dong, Zhiyong Yang

**Affiliations:** ^1^Department of Neurosurgery, Shengjing Hospital of China Medical University, Shenyang, China; ^2^Department of Cardiology, Shengjing Hospital of China Medical University, Shenyang, China

**Keywords:** dietary fiber, stroke, myocardial infarction, cross-sectional study, NHANES

## Abstract

This study aimed to detect dietary fiber intake and its association with nonfatal cardiovascular/cerebrovascular events (myocardial infarction and stroke) in adults in the United States. This cross-sectional study obtained data from the 2011–2018 National Health and Nutrition Examination Survey database. Using multivariate logistic regression, we compared dietary fiber intake across demographics and detected an association between dietary fiber intake and patient-reported nonfatal myocardial infarction and/or stroke events. We enrolled 8,872 participants (mean dietary fiber intake, 17.38 ± 0.22 g/day). The weighted prevalence of nonfatal cardiovascular/cerebrovascular events was 5.36%, which decreased with higher dietary fiber intake (nonfatal cardiovascular/cerebrovascular events: Tertile1, 6.50%; Tertile2, 5.45%; Tertile3, 4.25%). Higher fiber intake indicated a stable negative association with nonfatal cardiovascular/cerebrovascular events in the multivariate logistic regression analysis, weighted generalized additive model, and smooth curve fitting. Interaction tests showed no significant effect of demographic, socioeconomic, and disease status on the association between dietary fiber intake and nonfatal cardiovascular/cerebrovascular events. Dietary fiber intake was far below the recommended amount. Higher dietary fiber intake was associated with a lower prevalence of nonfatal cardiovascular/cerebrovascular events.

## Introduction

Cardiovascular and cerebrovascular diseases remain the major cause of death in the United States, and stroke and myocardial infarction (MI) are major cardiovascular and cerebrovascular disease events (1–3). Stroke and MI account for ~25 and 16.5% of cases of mortality from cardiovascular and cerebrovascular diseases, respectively. Stroke and MI are life-threatening conditions that account for more than 800,000 deaths annually in the United States, which makes them important public health issues. The estimated annual cost of stroke and MI management is ~$18 billion and $11.5 billion, respectively in the United States, which has a significant socioeconomic impact and burdens the healthcare system (4, 5). Therefore, it is necessary to identify preventable and controllable factors to reduce the incidence of cardiovascular and cerebrovascular diseases.

Dietary fiber is a type of carbohydrate that cannot be digested by endogenic digestive enzymes in the human body. It is recommended that dietary fiber intake be increased to confer beneficial health effects to humans (6). Observational studies and randomized control trials have found that increased dietary fiber intake contributes to lowering blood total cholesterol, low-density lipoprotein cholesterol, lipids, metabolic syndrome, glucose, and hypertension, which are known risk factors for cardiovascular and cerebrovascular diseases (7–12). Several cohort studies have revealed the benefits of dietary fiber in reducing the risk of cardiovascular and cerebrovascular diseases, including hemorrhagic stroke, ischemic stroke, and coronary heart disease (13–17). However, over the last decades, studies regarding dietary fiber with large sample sizes from the United States were limited and did not fully investigate the potential demographic and socioeconomic disparities and its effect on the correlation between dietary fiber intake and cardiovascular and cerebrovascular diseases among diverse ethnic groups. Moreover, our eating habits have changed greatly, and more ultra-processed foods that lack dietary fiber are being consumed from the last few decades. Therefore, the beneficial effect of dietary fiber on cardiovascular and cerebrovascular diseases needs to be reassessed. Hence, we conducted a large cross-sectional study to detect the potential association between dietary fiber intake and cardiovascular and cerebrovascular diseases, based on surveillance data from the National Health and Nutrition Examination Survey (NHANES) 2011–2018. We hypothesized that increased dietary fiber intake would be associated with a lower prevalence of nonfatal cardiovascular/cerebrovascular events.

## Materials and Methods

### Study Population

**Figure 1:**
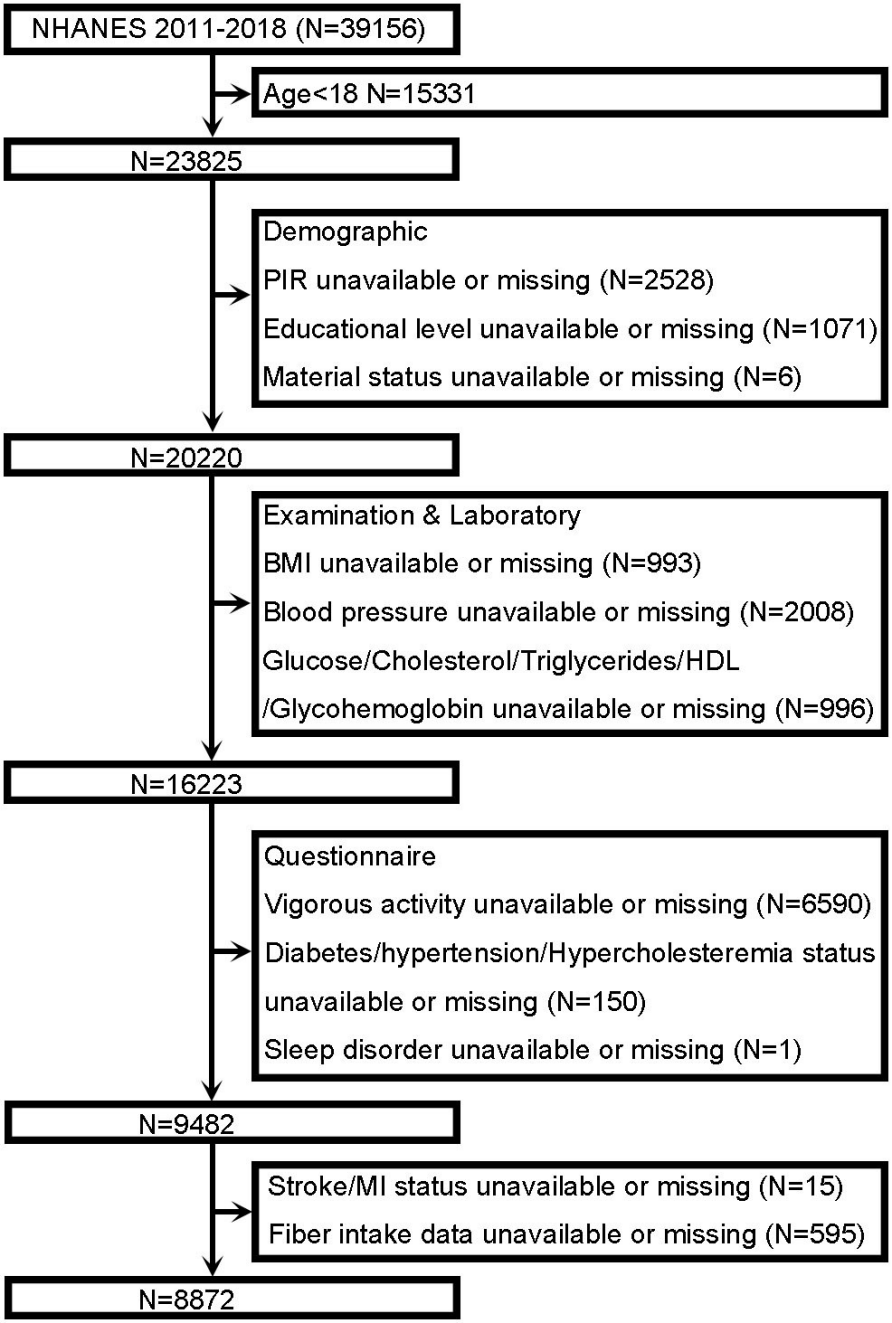
Flowchart of the participant from the National Health and Nutrition Examination Survey (NHANES) 2011–2018. PIR, poverty to income ratio; BMI, body mass index; HDL, high-density lipoprotein; MI, myocardial infarction.

We obtained data from the NHANES database, conducted by the National Center for Health Statistics of the Centers for Disease Control and Prevention of the United States, to evaluate the health and nutrition of the US population. A stratified multistage probability sampling method was adopted to achieve the representativeness of Americans. The NHANES begins with an at-home interview in which trained staff asks questions and automated data are collected. Subsequently, all of the participants visit a mobile examination clinic (MEC), where qualified personnel gather anthropometric data and biological samples. For this analysis, four cycles of NHANES (2011–2012, 2013–2014, 2015–2016, 2017–2018) with independent samples were utilized to accumulate an appropriate sample size. Individuals were excluded if they were younger than 18 years (*n* = 15,331) or responded with missing values for key analysis variables. The inclusion criteria in this study are summarized in the flowchart shown in Figure 1. The total number of individuals with missing variables for each study year is shown in Supplementary Figure S1, with no significant differences between them.

NHANES research has been approved by the Ethics Review Committee of the National Center for Health Statistics and Research. All participants in the survey signed the informed consent form. Access to NHANES database does not require ethical or administrative permission.

### Exposure and Outcome Definitions

Similar to earlier studies (18, 19), this study used the first 24-h dietary recall conducted by trained food recall data collectors at the MEC. The 24-h dietary data were linked to the US Department of Agriculture Food Surveys Nutrient Database for the estimation of energy and nutrient intake by the National Center for Health Statistics. The total intake of fiber was calculated by summing the amounts from food and supplemental sources.

We determined the outcomes using a Medical Condition Questionnaire. When a participant answered “yes” to the question “has a doctor ever told you that you had a heart attack,” we considered that he or she had MI. Similarly, when a participant answered “yes” to the question “has a doctor ever told you that you had a stroke,” we considered that he or she had a stroke (20, 21). Self-reported stroke and MI measures have been used in previous epidemiological studies using NHANES data, and results of several studies have revealed the self-reported measurement method is reliable (21–24). Outcomes included patient-reported nonfatal MI and/or stroke.

### Covariates

Based on the extant literature and clinical experience, variables with potential associations with fiber intake and nonfatal cardiovascular/cerebrovascular events were collected in this study. Covariates included sex (men/women); race (Mexican American, other races, non-hispanic White, or non-hispanic Black); marital status (married/living with partner, widowed/divorced/separated, or never married); educational level (< high school, high school, or >high school); poverty to income ratio (PIR; <1.2 or ≥1.2); energy intake (kcal/day); body mass index (BMI; <25 kg/m^2^, 25–30 kg/m^2^, or ≥30 kg/m^2^); current smoker (yes, no, or unknown); diabetes (yes, no, or borderline); hypertension (yes or no); hypercholesterolemia (yes or no); sleep disorder (yes or no); hypoglycemic drugs (yes, no, or unknown); antihypertensive drugs (yes, no, or unknown); lipid-lowering drugs (yes, no, or unknown); and preventive aspirin drugs (yes, no, or unknown). Height and weight were measured by an NHANES examiner, and BMI was calculated as weight (kg) divided by height (meters squared). Participants were assigned to morning, afternoon, or evening sessions before arriving at the MEC. Participants in the morning sessions fasted for at least 9 h; those in the afternoon or evening sessions consumed anything they wanted. Blood was collected by a certified phlebotomist according to the applicable standards and NHANES methodology. In brief, cholesterol (mg/dl) and high-density lipoprotein (mg/dl) levels were measured using the cholesterol oxidase method. Glucose (mg/dl) level was measured using the hexokinase method. Triglyceride (mg/dl) level was measured of hydrolysis of triglycerides by lipoprotein lipase. Glycohemoglobin (%) level was measured using the high-performance liquid chromatography method. For further information on methodology, refer to the Laboratory Method Files section for detailed laboratory procedure manual(s) of the methods (http://www.cdc.gov/nchs/nhanes.htm). Three systolic blood pressure (mmHg) and diastolic blood pressure (mmHg) readings were obtained for each participant, and the mean value was calculated using the three measurements. Vigorous activity (yes or no) was characterized by a significant increase in breathing or heart rate (involving carrying or lifting heavy loads, digging or construction work) for at least 10 min continuously (25, 26).

### Statistical Analyses

All statistical analyses were performed based on the Center for Disease Control and Prevention guideline. A complex multistage cluster surgery design analysis was considered, and an appropriate NHANES sample weight was applied. Continuous variables are presented as mean ± standard error, and categorical variables are presented as percentages. The weighted linear regression model (for continuous variables) or the weighted chi-squared test (for categorical variables) was utilized to analyze the intergroup difference index divided by fiber intake (Tertiles 1–3). Univariate and multivariate logistic regression analyses were used to explore the association between fiber intake and nonfatal cardiovascular/cerebrovascular events. In multivariate logistic regression, model 1 was adjusted for no covariates; model 2 was adjusted for age, sex, and race; and model 3 was adjusted for all covariates. A weighted generalized additive model and smooth curve fitting were utilized to further explore the association between fiber intake and nonfatal cardiovascular/cerebrovascular events. Subgroup analysis was performed by multivariate logistic regression stratified by age, sex, race, educational level, marital status, PIR, BMI, smoking status, vigorous activity, diabetes, hypertension, hypercholesterolemia, and sleeping disorders. An interaction term was also used to explore the heterogeneity of the association between different subgroups using the log-likelihood ratio test model. We performed all the analyses using Empower software (www.empowerstats.com; X&Y solutions, Inc., Boston, MA, USA) and R version 3.4.3 (http://www.Rproject.org, The R Foundation) (27–29).

## Results

### Baseline Characteristics of the Participants

The weighted population baseline characteristics of the participants are demonstrated in Table 1. A total of 8,872 participants were recruited in this study, of which 51.57% were female and 48.43% were male, with a mean age of 47.64 ± 0.39 years. Regarding races, 7.79, 14.02, 67.81, and 10.38% of the participants were Mexican American, had other races, were non-hispanic White, and were non-hispanic Black, respectively. Moreover, 63.67, 18.18, and 18.16% of the participants were married/living with partner, widowed/divorced/separated, and never married, respectively. Furthermore, the participants had the following educational levels: 12.56%, < high school; 21.72%, high school; and 65.72%, >high school. Overall, the PIR of 18.51% of the participants was <1.2. The average fiber intake was 17.38 ± 0.22 g/day. Participants were divided into tertiles according to dietary fiber intake levels (Tertile1, 7.51 ± 0.07 g/day; Tertile2, 14.90 ± 0.05 g/day; and Tertile3, 28.79 ± 0.25 g/day). The overall weighted prevalence of nonfatal cardiovascular/cerebrovascular events was 5.36%, and the participants in the higher fiber intake tertile had a lower prevalence of nonfatal cardiovascular/cerebrovascular events than the participants in the lower fiber intake tertile (Tertile1, 6.50%; Tertile2, 5.45%; and Tertile3, 4.25%; Table 1).

**Table 1:** Baseline characteristics of the participants, weighted.

**Characteristic**	**Fiber intake, g/day**		
	**Tertile1 (7.51 ±0.07 g/day)**	**Tertile2 (14.90 ±0.05 g/day)**	**Tertile3 (28.79 ±0.25 g/day)**
Age (years)	46.88 ± 0.49	48.14 ± 0.52	47.82 ± 0.57
**Sex**, ***n*** **(%)**			
Male	40.01	46.22	58.27
Female	59.99	53.78	41.73
**Race**, ***n*** **(%)**			
Mexican American	5.34	7.36	10.41
Other races	13.48	12.78	15.77
Non-hispanic White	66.89	70.22	66.21
Non-hispanic Black	14.30	9.65	7.61
**Marital status**, ***n*** **(%)**			
Married/living with partner	58.55	63.72	68.22
Widowed/divorced/separated	21.54	19.43	13.88
Never married	19.92	16.85	17.90
**Educational level**, ***n*** **(%)**			
< High school	15.32	11.73	10.91
High school	25.99	22.87	16.70
>High school	58.69	65.40	72.39
**PIR**, ***n*** **(%)**			
<1.2	23.50	16.29	16.28
≥1.2	76.50	83.71	83.72
**BMI**, ***n*** **(%)**			
<25 kg/m^2^	26.70	28.09	30.65
25–30 kg/m^2^	30.16	33.28	35.00
≥30 kg/m^2^	43.14	38.63	34.35
**Current smoker**, ***n*** **(%)**			
Yes	8.21	5.46	4.82
No	5.36	6.76	9.04
Unknown	86.43	87.78	86.14
Systolic blood pressure (mmHg)	122.59 ± 0.41	121.78 ± 0.42	121.90 ± 0.44
Diastolic blood pressure (mmHg)	70.31 ± 0.41	70.70 ± 0.36	70.97 ± 0.38
Glucose (mg/dl)	99.78 ± 0.91	99.41 ± 0.69	99.50 ± 0.82
Cholesterol (mg/dl)	192.35 ± 0.97	193.18 ± 1.07	192.18 ± 1.12
Triglycerides (mg/dl)	147.47 ± 2.77	149.05 ± 3.20	151.26 ± 3.59
HDL (mg/dl)	54.02 ± 0.55	54.84 ± 0.48	53.47 ± 0.48
Glycohemoglobin (%)	5.62 ± 0.02	5.63 ± 0.02	5.63 ± 0.03
Energy intake (kcal/day)	1,601.75 ± 16.63	2,145.46 ± 21.92	2,742.15 ± 25.63
**Vigorous activity**, ***n*** **(%)**			
Yes	23.21	23.02	22.73
No	76.79	76.98	77.27
**Diabetes**, ***n*** **(%)**			
Yes	10.05	9.54	8.94
No	87.09	88.56	88.64
Borderline	2.86	1.90	2.42
**Hypertension**, ***n*** **(%)**			
Yes	32.78	33.47	31.10
No	67.22	66.53	68.90
**Hypercholesteremia**, ***n*** **(%)**			
Yes	32.75	34.77	35.28
No	67.25	65.23	64.72
**Sleeping disorder**, ***n*** **(%)**			
Yes	31.85	29.97	27.71
No	68.15	70.03	72.29
**Hypoglycemic drugs**, ***n*** **(%)**			
Yes	7.01	8.16	7.09
No	12.59	10.39	11.70
Unknown	80.39	81.45	81.21
**Antihypertensive drugs**, ***n*** **(%)**			
Yes	23.39	25.12	21.76
No	5.04	3.98	4.34
Unknown	71.57	70.90	73.90
**Lipid-lowing drugs**, ***n*** **(%)**			
Yes	18.30	18.75	17.75
No	5.71	5.77	6.53
Unknown	75.99	75.48	75.72
**Preventive aspirin use**, ***n*** **(%)**			
Yes	2.03	2.52	3.36
No	39.63	38.61	39.07
Unknown	58.35	58.86	57.57
**Nonfatal cardiovascular event**, ***n*** **(%)**			
Yes	6.50	5.45	4.25
No	93.50	94.55	95.75

*Mean ± standard error (SE) for continuous variables and percentage (%) for categorical variables*.

*PIR, poverty to income ratio; BMI, body mass index; HDL, high-density lipoprotein*.

### Higher Dietary Fiber Intake Was Associated With Lower Prevalence of Nonfatal Cardiovascular Event

**Figure 2:**
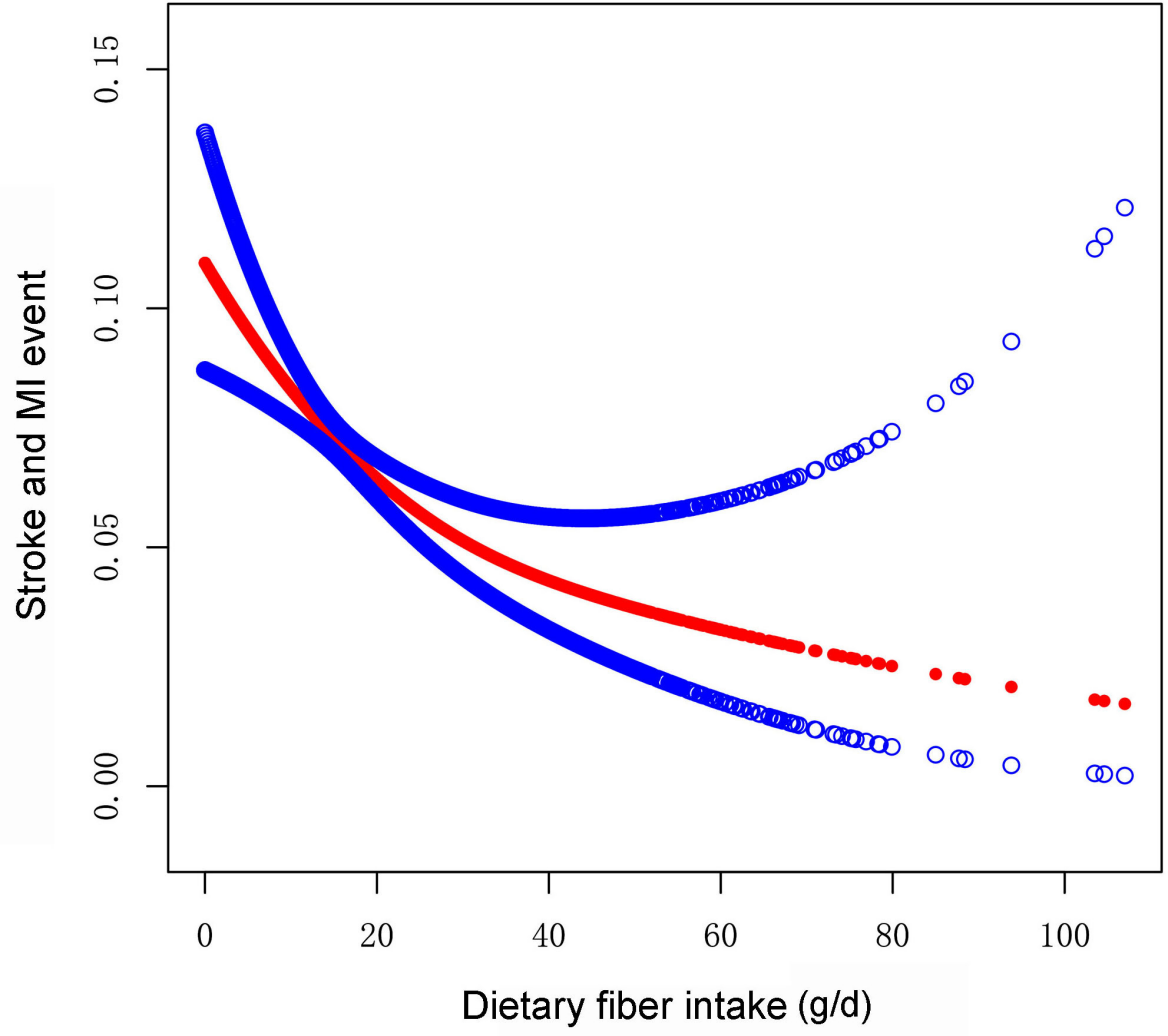
Association between dietary fiber intake and odds of nonfatal cardiovascular event (stroke and MI) using the generalized additive model. Adjusting for potential confounding variables (age; sex; race; marital status; educational level; PIR; BMI; smoking status; systolic blood pressure; diastolic blood pressure; glucose, cholesterol, triglyceride, HDL, and glycohemoglobin levels; energy intake; vigorous activity; diabetes; hypertension; hypercholesterolemia; sleeping disorder; and hypoglycemic, antihypertensive, lipid-lowing, and aspirin drugs). The red points line represents the fitting spline. The blue points line represents the 95% confidence intervals. MI, myocardial infarction.

First, we performed univariate logistic regression analysis (Table 2). Participants with higher dietary fiber intake were 2% less likely to have nonfatal cardiovascular/cerebrovascular events than those with lower dietary fiber intake [odds ratio (OR) = 0.98; 95% confidence interval (CI), 0.97–0.99]. Negative associations were still observed when we converted fiber intake from a continuous variable to a categorical variable (tertiles). Compared with Tertile1, participants with higher intake of dietary fiber were 17 and 36% less likely to have nonfatal cardiovascular/cerebrovascular events in Tertile2 (OR = 0.83; 95% CI, 0.66–1.04) and Tertile3 (OR = 0.64; 95% CI, 0.48–0.84; Table 2). Furthermore, in multivariate logistic regression analysis, higher fiber intake was associated with lower odds of nonfatal cardiovascular/cerebrovascular events. The OR and 95% CI in model 1, which did not adjust for covariates, were 0.98 and 0.97–0.99, respectively. The OR and 95% CI in model 2, which was adjusted for age, sex, and race, were 0.97 and 0.96–0.99, respectively. The OR and 95% CI in model 3, which was adjusted for all the covariates, were 0.98 and 0.96–1.00, respectively. In the sensitivity analysis, the adjusted ORs (reference to Tertile1) were 0.81 (95% CI, 0.61–1.09) for Tertile 2 and 0.64 for Tertile 3 (95% CI, 0.46–0.91) in model 3 (Table 3). We also used a generalized additive model and smooth curve fitting to further detect the association between fiber intake and nonfatal cardiovascular/cerebrovascular events, which showed that fiber intake was negatively associated with nonfatal cardiovascular/cerebrovascular events (Figure 2). To avoid some significantly extreme fiber intake affecting the results, extreme fiber intakes, defined as values with more than 3 standard deviations from the mean (*n* = 131), were removed from the analysis. Multivariate logistic regression analysis (Supplementary Table S1) and smooth curve fitting (Supplementary Figure S2) also suggested a negative association between fiber intake and nonfatal cardiovascular/cerebrovascular events. The above analysis suggested a stable negative association between higher fiber intake and odds of nonfatal cardiovascular/cerebrovascular events.

**Table 2:** Results of the univariate logistic regression analysis of factors associated with nonfatal cardiovascular/cerebrovascular events, weighted.

	**Statistics**	**OR (95%CI)**
Age (years)	47.64 ± 0.39	1.08 (1.07, 1.09)
**Sex**, ***n*** **(%)**		
Male (*n* = 4,370)	48.43	Ref
Female (*n* = 4,502)	51.57	0.71 (0.56, 0.90)
**Race**, ***n*** **(%)**		
Mexican American (*n* = 1,129)	7.79	Ref
Other races (*n* = 2,248)	14.02	1.50 (1.01, 2.23)
Non-hispanic White (*n* = 3,545)	67.81	1.71 (1.19, 2.47)
Non-hispanic Black (*n* = 1,950)	10.38	2.01 (1.44, 2.81)
**Marital status**, ***n*** **(%)**		
Married/Living with partner (*n* = 5,228)	63.67	Ref
Widowed/divorced/separated (*n* = 1,936)	18.18	1.95 (1.54, 2.47)
Never married (*n* = 1,708)	18.16	0.44 (0.30, 0.64)
**Educational level**, ***n*** **(%)**		
< High school (*n* = 1,738)	12.56	Ref
High school (*n* = 1,944)	21.72	0.88 (0.65, 1.19)
>High school (*n* = 5,190)	65.72	0.45 (0.33, 0.60)
**PIR**, ***n*** **(%)**		
<1.2 (*n* = 2,442)	18.51	Ref
≥1.2 (*n* = 6,430)	81.49	0.72 (0.57, 0.91)
**Bady mass index**, ***n*** **(%)**		
<25 kg/m^2^ (*n* = 2,554)	28.54	Ref
25 to 30 kg/m^2^ (*n* = 2,874)	32.91	1.28 (0.95, 1.72)
≥30 kg/m^2^ (*n* = 3,444)	38.56	1.89 (1.41, 2.53)
**Current smoker**, ***n*** **(%)**		
Yes (*n* = 546)	6.09	Ref
No (*n* = 583)	7.11	1.32 (0.66, 2.65)
Unknown (*n* = 7,743)	86.80	0.86 (0.52, 1.40)
Systolic blood pressure (mmHg)	122.07 ± 0.27	1.02 (1.01, 1.03)
Diastolic blood pressure (mmHg)	70.67 ± 0.30	0.98 (0.97, 0.99)
Glucose (mg/dl)	99.55 ± 0.48	1.01 (1.01, 1.01)
Cholesterol (mg/dl)	192.58 ± 0.69	0.99 (0.99, 0.99)
Triglycerides (mg/dl)	149.32 ± 2.28	1.00 (1.00, 1.00)
HDL (mg/dl)	54.12 ± 0.35	0.99 (0.98, 1.00)
Glycohemoglobin (%)	5.63 ± 0.02	1.45 (1.36, 1.55)
Energy intake (kcal/day)	2,182.17 ± 10.89	1.00 (1.00, 1.00)
**Vigorous activity**, ***n*** **(%)**		
Yes (*n* = 1,840)	22.98	Ref
No (*n* = 7,032)	77.02	1.42 (1.03, 1.97)
**Diabetes**, ***n*** **(%)**		
Yes (*n* = 1,143)	9.49	Ref
No (*n* = 7,499)	88.13	0.20 (0.15, 0.26)
Borderline (*n* = 230)	2.38	0.62 (0.35, 1.09)
**Hypertension**, ***n*** **(%)**		
Yes (*n* = 3,228)	32.44	Ref
No (*n* = 5,644)	67.56	0.19 (0.15, 0.24)
**Hypercholesteremia**, ***n*** **(%)**		
Yes (*n* = 3,107)	34.32	Ref
No (*n* = 5,765)	65.68	0.23 (0.18, 0.30)
**Sleeping disorder**, ***n*** **(%)**		
Yes (*n* = 2,403)	29.77	Ref
No (*n* = 6,469)	70.23	0.57 (0.46, 0.71)
**Hypoglycemic drugs**, ***n*** **(%)**		
Yes (*n* = 874)	7.44	Ref
No (*n* = 1,125)	11.52	0.65 (0.45, 0.93)
Unknown (*n* = 6,873)	81.04	0.21 (0.15, 0.28)
**Antihypertensive drugs**, ***n*** **(%)**		
Yes (*n* = 2,426)	23.44	Ref
No (*n* = 396)	4.43	0.41 (0.23, 0.71)
Unknown (*n* = 6,050)	72.13	0.16 (0.12, 0.20)
**Lipid-lowing drugs**, ***n*** **(%)**		
Yes (*n* = 1,749)	18.27	Ref
No (*n* = 584)	6.01	0.32 (0.21, 0.50)
Unknown (*n* = 6,539)	75.72	0.11 (0.09, 0.14)
**Preventive aspirin use**, ***n*** **(%)**		
Yes (*n* = 190)	2.66	Ref
No (3,437)	39.08	1.08 (0.33, 3.57)
Unknown (*n* = 5,245)	58.26	3.39 (1.08, 10.65)
Fiber intake (g/day)	17.38 ± 0.22	0.98 (0.97, 0.99)
**Fiber intake tertile**, ***n*** **(%)**		
Tertile1 (*n* = 2,929)	30.88	Ref
Tertile2 (*n* = 2,959)	34.82	0.83 (0.66, 1.04)
Tertile3 (*n* = 2,984)	34.29	0.64 (0.48, 0.84)

*Mean ± standard error (SE) for continuous variables, Percentage (%) for categorical variables. p-Value was calculated via logistic regression analysis*.

*Tertile1, 7.51 ± 0.07 g/day; Tertile2, 14.90 ± 0.05 g/day; Tertile3, 28.79 ± 0.25 g/day*.

*OR, odds ration; 95% CI, 95% confidence interval; PIR, poverty to income ratio; BMI, body mass index; HDL, high density lipoprotein*.

**Table 3:** Results of the multivariate logistic regression analysis of association between fiber intake and nonfatal cardiovascular/cerebrovascular events, weighted.

**Variable**	**Model1^a^ [OR (95%CI)]**	**Model2^b^ [OR (95%CI)]**	**Model3^c^ [OR (95%CI)]**
Fiber intake (g/day)	0.98 (0.97, 0.99)	0.97 (0.96, 0.99)	0.98 (0.96, 1.00)
**Fiber intake (Tertile)**, ***n*** **(%)**			
Tertile1 (*n* = 2,929)	Ref	Ref	Ref
Tertile2 (*n* = 2,959)	0.83 (0.66, 1.04)	0.76 (0.59, 0.96)	0.81 (0.61, 1.09)
Tertile3 (*n* = 2,984)	0.64 (0.48, 0.84)	0.56 (0.42, 0.76)	0.64 (0.46, 0.91)

*In sensitivity analysis, fiber intake was converted from a continuous variable to a categorical variable (tertile). OR, odds ratio; 95% CI, 95% confidence interval*.

*Tertile1, 7.51 ± 0.07 g/day; Tertile2, 14.90 ± 0.05 g/day; and Tertile3, 28.79 ± 0.25 g/day*.

a *Model 1: Adjusted for no covariates*.

b *Model 2: Adjusted for age, sex, and race*.

c *Model 3: Adjusted for age; sex; race; marital status; educational level; PIR; BMI; smoking status; systolic blood pressure; diastolic blood pressure; glucose, cholesterol, triglyceride, HDL, and glycohemoglobin levels; energy intake; vigorous activity; diabetes; hypertension; hypercholesterolemia; sleeping disorder; and hypoglycemic, antihypertensive, lipid-lowing, and aspirin drugs*.

### Subgroup Analysis

A subgroup analysis was performed to analyze the robustness of the association between fiber intake and nonfatal cardiovascular/cerebrovascular events. We analyzed the interactions with age, sex, marital status, race, educational level, PIR, BMI, smoking status, vigorous activity, diabetes, hypercholesterolemia, hypertension, and sleeping disorders. No dependence on the variates mentioned above was observed for the association between fiber intake and nonfatal cardiovascular/cerebrovascular events (all *p* for interaction >0.01) (Table 4). The results suggested that the negative association between fiber intake and nonfatal cardiovascular/cerebrovascular events was robust in populations with different demographic, socioeconomic, and disease status, and that it might be appropriate for various populations.

**Table 4:** Subgroup analysis of association between fiber intake and nonfatal cardiovascular/cerebrovascular events.

	**OR (95%CI)**	***p*** **for interaction**
**Stratified by age**		0.6653
Age <60 (*n* = 5,964)	0.98 (0.96, 1.01)	
Age≥60 (*n* = 2,908)	0.99 (0.97, 1.00)	
**Stratified by sex**		0.9767
Male (*n* = 4,370)	0.98 (0.96, 1.00)	
Female (*n* = 4,502)	0.98 (0.96, 1.00)	
**Stratified by race**		0.8813
Mexican American (*n* = 1,129)	0.98 (0.95, 1.01)	
Other races (*n* = 2,248)	0.98 (0.95, 1.02)	
Non-hispanic White (*n* = 3,545)	0.98 (0.96, 1.00)	
Non-hispanic Black (*n* = 1,950)	0.99 (0.96, 1.01)	
**Stratified by marital status**		0.7917
Married/Living with partner (*n* = 5,228)	0.97 (0.96, 0.99)	
Widowed/ Divorced/ Separated (*n* = 1,936)	0.99 (0.96, 1.02)	
Never married (*n* = 1,708)	0.98 (0.95, 1.01)	
**Stratified by educational level**		0.9634
< High school (*n* = 1,738)	0.98 (0.96, 1.01)	
High school (*n* = 1,944)	0.98 (0.95, 1.00)	
>High school (*n* = 5,190)	0.98 (0.96, 1.00)	
**Stratified by PIR**		0.3615
<1.2 (*n* = 2,442)	0.99 (0.96, 1.01)	
≥1.2 (*n* = 6,430)	0.98 (0.96, 0.99)	
**Stratified by BMI**		0.4485
<25 kg/m^2^ (*n* = 2,554)	0.98 (0.96, 1.01)	
25–30 kg/m^2^ (*n* = 2,874)	0.97 (0.94, 0.99)	
≥30 kg/m^2^ (*n* = 3,444)	0.99 (0.96, 1.01)	
**Stratified by smoke**		0.2253
Yes (*n* = 546)	0.98 (0.94, 1.03)	
No (*n* = 583)	0.95 (0.90, 0.99)	
Unknown (7,743)	0.98 (0.97, 1.00)	
**Stratified by vigorous activity**		0.9716
Yes (*n* = 1,840)	0.98 (0.95, 1.01)	
No (*n* = 7,032)	0.98 (0.96, 1.00)	
**Stratified by diabetes**		0.9645
Yes (*n* = 1,143)	0.98 (0.96, 1.00)	
No (*n* = 7,499)	0.98 (0.96, 1.00)	
Borderline (*n* = 230)	0.97 (0.90, 1.05)	
**Stratified by hypertension**		0.0364
Yes (*n* = 3,228)	0.99 (0.97, 1.01)	
No (*n* = 5,644)	0.96 (0.94, 0.98)	
**Stratified by hypercholesteremia**		0.0692
Yes (*n* = 3,107)	0.99 (0.97, 1.01)	
No (*n* = 5,765)	0.96 (0.94, 0.99)	
**Stratified by sleeping disorder**, ***n*** **(%)**		0.4559
Yes (*n* = 2,403)	0.99 (0.96, 1.01)	
No (*n* = 6,469)	0.97 (0.95, 1.00)	

*The results of the subgroup analysis were adjusted for all covariates (age; sex; race; marital status; educational level; PIR; BMI; smoking status; systolic blood pressure; diastolic blood pressure; glucose, cholesterol, triglyceride, HDL, and glycohemoglobin levels; energy intake; vigorous activity; diabetes; hypertension; hypercholesterolemia; sleeping disorder; and hypoglycemic, antihypertensive, lipid-lowing, and aspirin drugs), except for the corresponding subgroup variables*.

*PIR, poverty to income ratio; BMI, body mass index; OR, odds ratio; 95% CI, 95% confidence interval*.

## Discussion

A total of 8,872 participants were included for this study, and the weighted average of dietary fiber intake was 17.38 ± 0.22 g/day.

Dietary fiber intake was slightly higher compared with survey results for mean dietary fiber intake of 16.2 g/day from 1999–2010 NHANES data (30). The overall weighted prevalence of nonfatal cardiovascular/cerebrovascular events was 5.36%. The prevalence of stroke and MI in the highest fiber intake group (Tertile3) was 4.25%. As dietary fiber intake decreased from the Tertile2 to Tertile1 group, stroke and MI increased gradually from 5.45 to 6.50%. We further detected that higher dietary fiber intake was independently associated with an increased prevalence of stroke and MI in multivariate regression analysis. This association was robust in subgroups stratified by demographic, socioeconomic, and disease status. Changing the typical diet and increasing dietary fiber intake to generate the beneficial health effects to improve cardiovascular and cerebrovascular diseases in US adults are long-term tasks.

Although several studies have reported the benefits of dietary fiber in decreasing the risk of cardiovascular and cerebrovascular diseases, the average daily fiber intake is 17 g/day in the United States, which is significantly lower than the recommended amount. The recommended dietary reference intakes are 25 g/day and 38 g/day for women and men aged 19–50 years, respectively. For patients aged >51 years, the recommended dietary fiber intake is higher. The benefits of dietary fiber intake in lowering cholesterol and lipid levels were reported in South African Bantu for the first time in 1954 (31). A meta-analysis, including 10 cohort study with 6–10 years' follow-up, found that dietary fiber intake was inversely correlated with the risk of cardiovascular and cerebrovascular diseases [relative risk (RR) = 0.84; 95% CI, 0.70–0.99]; however, an increase of 10 g of fiber intake per day was not statistically significant, with an RR of 1.0 (95% CI, 0.88–1.13) (32). Dietary fiber includes soluble and insoluble fiber, which is the indigestible carbohydrates and lignin in plants. Soluble fibers include viscous fibers, such as gum, non-viscous fibers, pectin, fructans, and β-glucan, and insoluble fibers include cellulose, lignin, and hemicellulose (33, 34). Dietary fiber can increase food transit time, delay nutrient absorption, and increase the fecal bulk, which contribute to slowing the absorption of glucose, cholesterol, and lipids. Additionally, dietary fiber is fermented by gut bacteria and generates short-chain fatty acids, which can reduce cholesterol synthesis in the liver and blood (35–37). Increasing dietary fiber intake may reduce the incidence of cardiovascular and cerebrovascular diseases by ameliorating the risk factors, such as diabetes and dyslipidemia. Twenty years previously, a few studies showed that increased fiber intake exerted more protective effect in cardiovascular and cerebrovascular diseases (RR = 0.71; 95% CI, 0.47–0.95 for coronary heart disease; RR = 0.74; 95% CI, 0.63–0.86 for stroke) as compared to the results from our study (OR = 0.98; 95% CI, 0.96–1.00) (38). These results might be due to the fact that ultra-processed food consumption increased, which weakened the beneficial effect of dietary fiber on cardiovascular and cerebrovascular diseases.

Different ethnic and economic statuses may lead to different levels of dietary fiber intake due to different eating habits. Hispanic Black and lower family income populations were correlated with lower dietary fiber intake among adults (39). Our study showed that participants of different races and economic statuses could benefit from cardiovascular and cerebrovascular diseases by increasing dietary fiber levels, and more attention should be paid to these vulnerable groups. Previous observational and randomized controlled studies revealed that obesity, diabetes, hypertension, and hypercholesterolemia are risk factors for cardiovascular and cerebrovascular diseases. According to the subgroup analysis in our study, the negative association between fiber intake, stroke, and MI was stable in the subgroup stratified by BMI, hypertension, diabetes, and hypercholesterolemia. In addition, we did not find any dependence on age, sex, race, educational level, marital status, PIR, BMI, smoking status, vigorous activity, diabetes, hypertension, hypercholesterolemia, and sleeping disorders for this association (all *p* for interaction >0.01), which implied that this negative association may be appropriate for various population settings.

Our study has several strengths. Our study included a large number of samples from the NHANES database and was adjusted for multivariate confounding variables to ensure reliability of the results. The association between fiber intake and stroke and MI was adequately detected in diverse groups. The results were stable and robust, which could be applied to a wide range of populations and, hence, address the study gap regarding the effect of dietary fiber on cardiovascular and cerebrovascular diseases over the last decade. However, the limitations of this study cannot be ignored. First, we could only conclude an association and not a causal interference, owing to the cross-sectional nature of this study. Second, dietary fiber intake may not be accurately evaluated based on 24-h recall measures. Further, this study did not analyze the dietary fiber intake from different sources. Finally, although we recruited various confounding variables in the analysis, we could not exclude the confounding effects of excluded or unknown variables.

## Conclusions

We found that dietary fiber intake was far below the recommended amount in the United States from 2011 to 2018. Higher dietary fiber intake is associated with a lower prevalence of stroke and MI. We should advocate further increase in dietary fiber intake to confer benefits for cardiovascular and cerebrovascular diseases.

## Data Availability Statement

The original contributions presented in the study are included in the article/Supplementary Material, further inquiries can be directed to the corresponding author.

## Author Contributions

Conceptualization and formal analysis: WD and ZY. Methodology, software, investigation, data curation, and writing—original draft preparation: WD. Writing—review and editing and supervision: ZY. All authors contributed to the article and approved the submitted version.

## Conflict of Interest

The authors declare that the research was conducted in the absence of any commercial or financial relationships that could be construed as a potential conflict of interest.

## Publisher's Note

All claims expressed in this article are solely those of the authors and do not necessarily represent those of their affiliated organizations, or those of the publisher, the editors and the reviewers. Any product that may be evaluated in this article, or claim that may be made by its manufacturer, is not guaranteed or endorsed by the publisher.

## Supplementary Material

Supplementary Figure S1 Flowchart of the total number of individuals with missing variables per study year from National Health and Nutrition Examination Survey (NHANES) 2011–2018.

Supplementary Figure S2 Association between dietary fiber intake (± 3 SD above the mean excluded) and odds of nonfatal cardiovascular event (stroke and MI) using the generalized additive model. Adjusting for potential confounding variables (age; sex; race; marital status; educational level; PIR; BMI; smoking status; systolic blood pressure; diastolic blood pressure; glucose, cholesterol, triglyceride, HDL, and glycohemoglobin levels; energy intake; vigorous activity; diabetes; hypertension; hypercholesterolemia; sleeping disorder; and hypoglycemic, antihypertensive, lipid-lowing, and aspirin drugs). The red points line represents the fitting spline. The blue points line represents the 95% confidence intervals. MI, myocardial infarction.
