# Supplementary figures and images for: Association of Dietary Fiber Intake With Myocardial Infarction and Stroke Events in US Adults: A Cross-Sectional Study of NHANES 2011–2018
Source: Front Nutr. 2022 Jun 21;9:936926. doi: 10.3389/fnut.2022.936926 (PMC9253671; doi:10.3389/fnut.2022.936926)

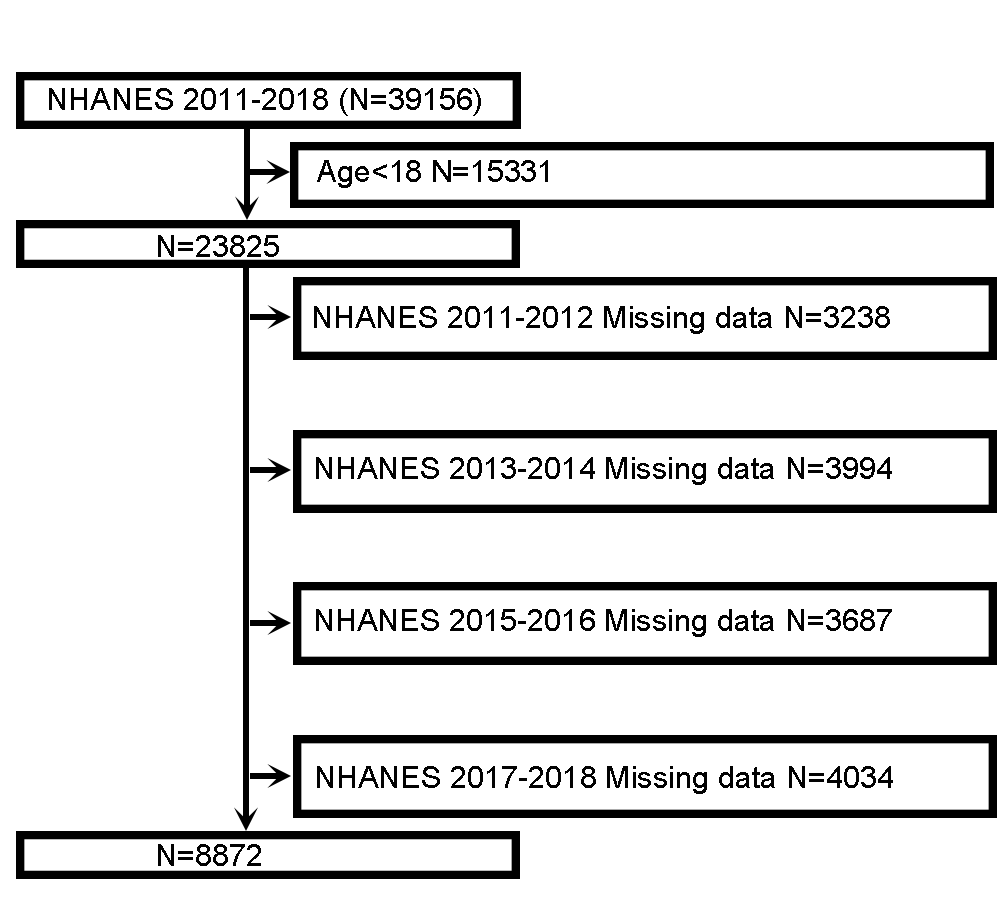

Supplement: Supplementary Figure S1 — Flowchart of the total number of individuals with missing variables per study year from National Health and Nutrition Examination Survey (NHANES) 2011–2018. [file Image_1.TIF]

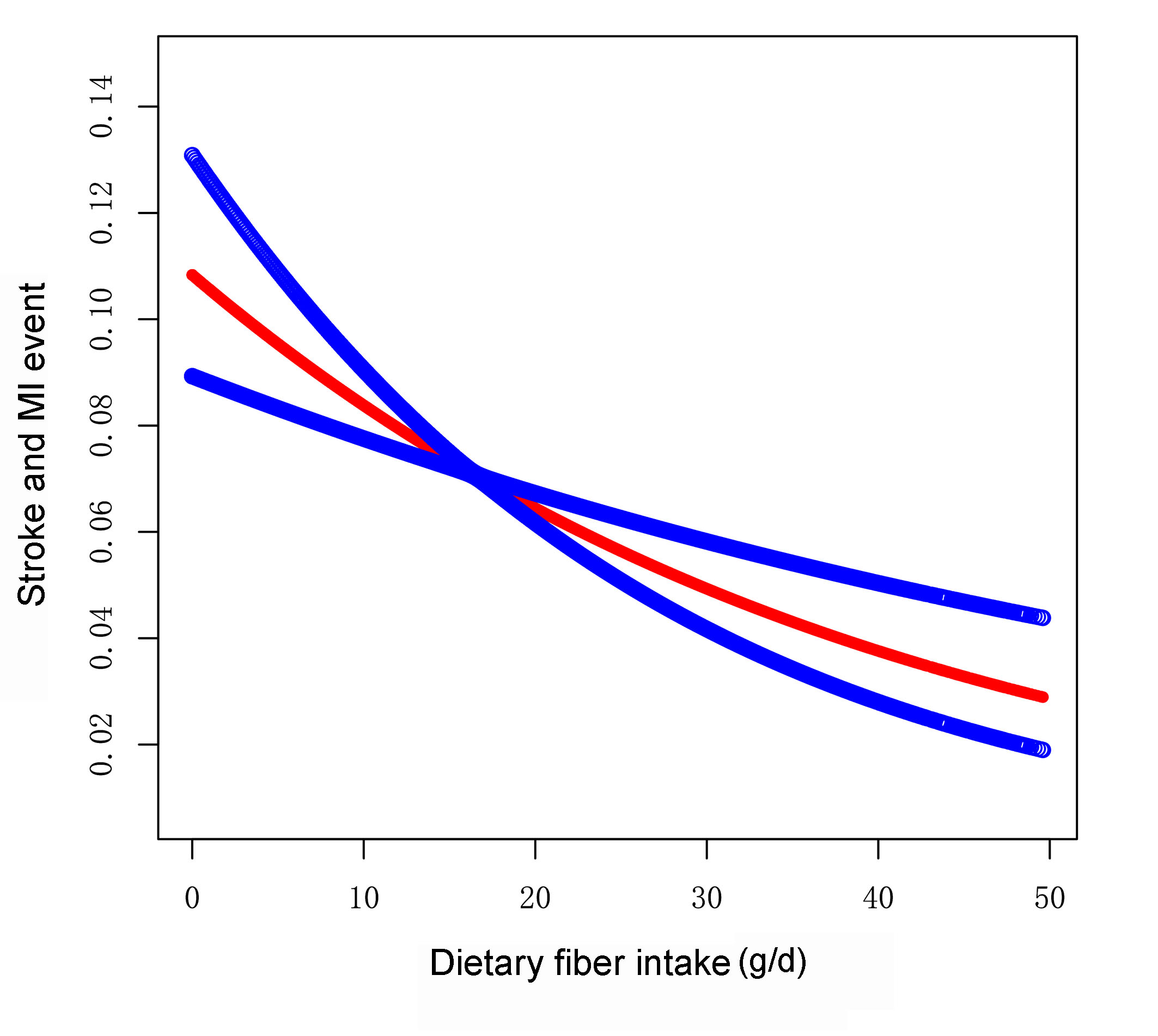

Supplement: Supplementary Figure S2 — Association between dietary fiber intake (± 3 SD above the mean excluded) and odds of nonfatal cardiovascular event (stroke and MI) using the generalized additive model. Adjusting for potential confounding variables (age; sex; race; marital status; educational level; PIR; BMI; smoking status; systolic blood pressure; diastolic blood pressure; glucose, cholesterol, triglyceride, HDL, and glycohemoglobin levels; energy intake; vigorous activity; diabetes; hypertension; hypercholesterolemia; sleeping disorder; and hypoglycemic, antihypertensive, lipid-lowing, and aspirin drugs). The red points line represents the fitting spline. The blue points line represents the 95% confidence intervals. MI, myocardial infarction. [file Image_2.jpg]
