# Supplementary material for: Association of Dietary Fiber Intake With Myocardial Infarction and Stroke Events in US Adults: A Cross-Sectional Study of NHANES 2011–2018
Source: Front Nutr. 2022 Jun 21;9:936926. doi: 10.3389/fnut.2022.936926 (PMC9253671; doi:10.3389/fnut.2022.936926)
Supplement: Supplementary file 3 [file Table_1.docx]

Table S1 Results of the multivariate logistic regression analysis of association between fiber intake (±3 SD above the mean excluded) and nonfatal cardiovascular/cerebrovascular events

| Variable | Model1^1^ (OR(95%CI)) | Model2^2^ (OR(95%CI)) | Model3^3^ (OR(95%CI)) |
| --- | --- | --- | --- |
| Fiber intake(g/d) | 0.97(0.96,0.99) | 0.97(0.95,0.98) | 0.97(0.95,0.99) |
| Fiber intake (Tertile) |  |  |  |
| Tertile1(n=2929) | Ref | Ref | Ref |
| Tertile2(n=2959) | 0.83(0.66,1.04) | 0.76(0.59,0.97) | 0.83(0.62,1.11) |
| Tertile3(n=2853) | 0.63(0.47,0.84) | 0.56(0.41,0.76) | 0.65(0.46,0.93) |

In sensitivity analysis, fiber intake was converted from a continuous variable to a categorical variable (tertile). OR, odds ratio; 95% CI, 95% confidence interval

Tertile1, 7.51±0.07 g/d; Tertile2, 14.90±0.05 g/d; and Tertile3, 27.51±0.19 g/d

^1^Model 1: Adjusted for no covariates

^2^Model 2: Adjusted for age, sex, and race

^3^Model 3: Adjusted for age; sex; race; marital status; educational level; PIR; BMI; smoking status; systolic blood pressure; diastolic blood pressure; glucose, cholesterol, triglyceride, HDL, and glycohemoglobin levels; energy intake; vigorous activity; diabetes; hypertension; hypercholesterolemia; sleeping disorder; and hypoglycemic, antihypertensive, lipid-lowing, and aspirin drugs
